# Systemic Treatment of Immune-Mediated Keratoconjunctivitis Sicca with Allogeneic Stem Cells Improves the Schirmer Tear Test Score in a Canine Spontaneous Model of Disease

**DOI:** 10.3390/jcm10245981

**Published:** 2021-12-20

**Authors:** Manuel Hermida-Prieto, Javier García-Castro, Luis Mariñas-Pardo

**Affiliations:** 1 Instituto de Investigación Biomédica de A Coruña—Universidade de A Coruña (INIBIC—UDC), 15006 A Coruna, Spain; manuel.hermida.prieto@sergas.es; 2 Faculty of Veterinary Medicine, Universidad Alfonso X El Sabio (UAX), 28691 Villanueva de la Canada, Spain; jgcastro@isciii.es; 3 Cellular Biotechnology Unit, Instituto de Salud Carlos III, 28029 Madrid, Spain

**Keywords:** keratoconjunctivitis sicca, mesenchymal stem cells, Schirmer tear test

## Abstract

Keratoconjunctivitis sicca (KCS) is characterized by ocular discomfort, conjunctival hyperaemia, and corneal scarring, causing reduced aqueous tear production that can be measured using the standard Schirmer tear test (STT). Canine adipose tissue-derived MSCs (cATMSCs) have been proposed as treatment due to their anti-inflammatory effect, by releasing cytokines and immunomodulatory soluble factors. Purpose: The aim of this study was to evaluate the effect of the systemic administration of cATMSCs on tear production in dogs with immune-mediated KCS, compared to classical Cyclosporine A (CsA) treatment. Methods: Twenty-eight client-owned dogs with spontaneous KCS were allocated in the experimental group (*n* = 14, treated with systemic cATMSCs or control group (*n* = 14, treated with CsA). SST values increased significantly at days 15 (*p* = 0.002), 45 (*p* = 0.042) and 180 (*p* = 0.005) with no observed side-effects in the experimental group. Eyes with an initial STT value of 11–14 mm/min maintained significant improvement at day 180, needing only artificial tears as treatment. Eyes with an initial STT value <11 mm/min needed cyclosporin treatment at day 45, so follow-up was stopped. Control animals treated with CsA did not improve their STT at day 180. Results and Conclusions: Systemic allogeneic cATMSCs application appeared to be a feasible and effective therapy with positive outcome in dogs with initial STT between 11–14 mm/min, with a significant improvement in tear production. The STT increment was maintained for at least 180 days, without needing additional medication, thus suggesting it could constitute an alternative therapy to classical immunosuppressive treatments.

## 1. Introduction

Companion dogs develop keratoconjunctivitis sicca (KCS), a disease that is very similar, immunopathologically and clinically, to humans’ dry eye syndrome (Sjogren’s syndrome) [1]. KCS is characterized by reduced aqueous tear production and inflammation, that can lead to ocular discomfort, conjunctival hyperemia, and corneal scarring. This condition can be evaluated using the standard Schirmer tear test (STT) strip [2]. It is often an under-recognized and/or a sub-clinical condition [3] which, in some breeds, is preceded by an immune-mediated destruction of lacrimal glands [4,5].

Immune-mediated KCS is the primarily accepted cause of KCS and the most prevalent of all possible forms [6,7]. Inflammation is characterized by lymphocyte infiltration and damage to the lacrimal gland, which diminishes tear production and consequently the aqueous tear film [5,8]. The ocular surface desiccates, gets inflamed, develops vascularization and scars in the cornea, with eventually vision lost in the dog [9,10].

Current therapies for immune-mediated KCS comprises immune-suppressive treatments [10,11] such as cyclosporine A (CsA) [12,13], tacrolimus [14,15], or pimecrolimus [15]. Short-term application of corticosteroids may improve symptoms but should be used with caution due to the occurrence of corneal ulceration [16], and immunosuppressive medication may include slight irritation of the eyes. Nevertheless, is the therapy is interrupted, the disease will go back to its initial status or even worsen. Besides, daily topical therapy causes low treatment adherence and the consequent lack of efficacy. Additionally, some cases do not respond to conventional medication [17].

For the above reasons, more specific and safer medications to treat inflammatory and immune-mediated ocular diseases needs to be developed. Mesenchymal stem cells (MSCs) are multipotent cells with the capacity to differentiate into diverse cell lineages and secrete different bioactive molecules with trophic, paracrine, and immunomodulatory functions [18,19,20]. The use of allogeneic MSCs is possible due to their low immunogenicity [21,22,23], allowing a fast therapy initiation without the need for harvesting MSCs from each patient.

Canine adipose tissue-derived MSCs (cATMSCs) have been used to treat dogs affected by bilateral dry eye disease by local implantation into the lacrimal gland and the gland of the third eyelids with positive outcomes [24,25]. It was proposed that cATMSCs act via an anti-inflammatory effect, by released proinflammatory cytokines and immunomodulatory soluble factors such as TGF-β [26,27,28].

MSCs’ local implantation needs to be performed by specifically trained staff, which limits the possible application in veterinary clinics. Less-invasive methods, such as intravenous administration, are preferable. Safety of the systemic administration of canine MSCs has been proven in bowel disease [29,30] or idiopathic autoimmune inflammatory disorders of the central nervous system [31] with excellent results.

In sum, the purpose of this study was to evaluate the effect of cATMSCs systemic administration on tear production in dogs with immune-mediated KCS.

## 2. Materials and Methods

### 2.1. Study Design

The study examined the systemic administration effects of cATMSCs in 14 client-owned dogs with KCS compared to a control group (*n* = 14) that received the standard treatment: CsAa three times a day (two drops at 1% solution) and artificial tears (Hylo Gel^®^). It was designed as a prospective, open-label controlled multicenter study developed at two veterinary facilities in Pontevedra and Oviedo (Spain). All animal procedures and protocols were conducted by licensed veterinary surgeons and complied with both national and European legislation for the protection of animals used for research experimentation and other scientific purposes (Spanish Royal Decree RD1386/2018 and EU Directive 2010/63/UE as modified by ECC/566/2015).

### 2.2. Isolation and Culture of cAdMSCs

cATMSCs were obtained and characterized as previously described [30]. Briefly, adipose tissue obtained from healthy donors was washed in Phosphate Buffered Saline 1X, minced and digested with collagenase NB4 Standard Grade, filtered and centrifuged until obtain the cell pellet. Primary cultures were carried out at 37 °C and 5% CO_2_ with Dulbecco’s modified Eagle’s medium containing 10% foetal bovine serum and 1% of glutamine-penicillin-streptomycin solution. Cells were detached when confluence was over 75% and subcultured at a concentration of 10^4^ cells/cm^2^ for continued passaging until passage 4 (P4). Cells were cryopreserved in vials and stored in liquid nitrogen until packaging for sending to the clinics.

### 2.3. cATMSCs Characterization by Flow Cytometry Analysis and In Vitro Multilineage Cell Differentiation 

Fluorescence-activated flow cytometry was used to characterize cATMSCs at P4 as previously described, [29] analyzing CD29, CD44, and CD90 as positive surface markers, and MHC-II, CD3, CD11, CD14, CD34, CD45, and CD79a as negative surface markers.

To assess the multipotentiality, cATMSCs at P4 were in vitro differentiated along adipogenic, osteogenic, and chondrogenic lineages according to standard protocols, as previously described [29,30].

### 2.4. Inclusion Criteria 

Client-owned dogs were included in this study. All animals suffered from spontaneous severe, moderate/mild, or early/subclinical KCS (STT ≤ 14 mm/min) for at least 12 months prior to recruitment. They were selected based on their clinical histories and fulfilment of, at least, one of the following symptoms: presence of mucoid ocular discharge or mucopurulent, corneal invasion, overlying epithelium, limitation of closing palpebral, subepithelial oedema, abrasions/ulcers, blepharospasm, pigmentary keratitis, keratohelcosis, and/or evaluation of three sectors of the ocular surface with Rose Bengal ˃3.5.

No restriction according to sex or breed was done. Dogs with evidence of corneal ulceration, infection processes, and tumors were not included. All dogs’ owners signed a written consent.

### 2.5. Prohibited and Allowed Medications and Therapies for Experimental Group

Any anti-inflammatory or immunomodulatory medications were withdrawn two weeks before cell therapy and suspended during the study follow-up. When additional treatment was necessary for animal welfare reasons, the animal was withdrawn from the study. The same diet, hygiene protocol, and use of a flea insecticidal medication was maintained during the study. Dogs were permitted to receive artificial tears in the follow-up under the specialist criterion.

### 2.6. Clinical Evaluation

**Figure 1:**
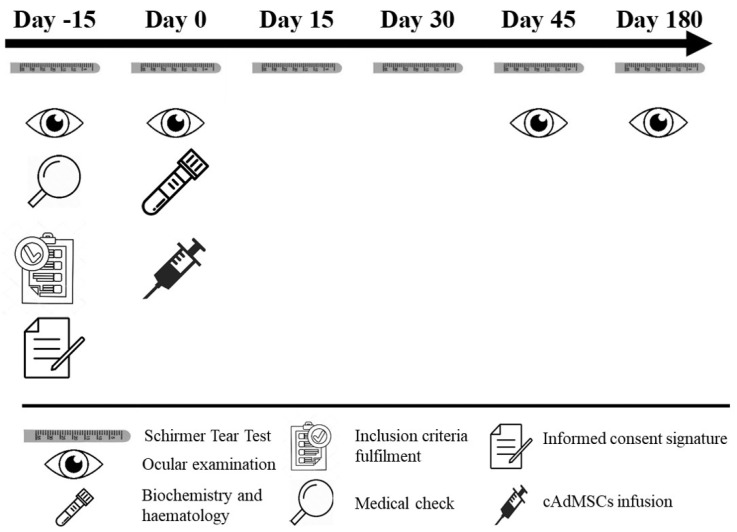
Schematic representation of the study from day −15 (recruitment) to day 180 of follow-up. Procedures performed are indicated at each study day. The key for the pictures is explained below. Legend: cAdMSC, canine adipose tissue-derived mesenchymal stem cell.

Clinical evaluations for each animal were performed by the same clinician. Dogs were evaluated before cell therapy administration and at days 0 (treatment), 15 ± 2, 30 ± 2, 45 ± 2 and 180 ± 2 after treatment. The study design is illustrated in Figure 1.

Each animal was given a full ophthalmoscopic examination using direct and indirect ophthalmoscopy. Type of conjunctivitis, conjunctival characterization, ocular surface evaluation, hematologic and serum biochemistry profile were obtained at day 0. Type of conjunctivitis was classified as transparent serous, mucoid or muco-purulent based on the veterinary expert criteria. Conjunctivitis characterization was assessed according a clinical scoring system (scale from 0 to 10) with respect to ocular symptoms based on the veterinary expert criteria. The ocular surface signs assessed were: corneal vascularization, overlaying epithelium, palpebral closing limitation, subepithelial oedema, abrasions/ulcers, blepharospasm, pigmentary keratitis, and keratohelcosis. The symptoms were classified as normal (0–1), mildly affected (2–4), moderately affected (5–7), and severely affected (8–10).

Ocular surface damage in the nasal conjunctiva, the temporal conjunctiva, and the cornea was graded as absent (0), mild (1), moderate (2), or severe (3) based on the results obtained after Rose Bengal staining. Total ocular surface damage result was the addition of the three previous values (0–9).

Tears production was measured using the STT at one minute (Schirmer I–without employing anesthetic) [31] with the following possible readings in mm/minute: 6 ≤ 5 insufficient tear production or severe KCS, 6–10 moderate/mild KCS, 11–14 early/subclinical KCS and ≥15 normal tear production.

### 2.7. cATMSCs Administration

Treatment dose was as follows: 10 × 10^6^ cATMSCs if body weight ≤10 kg, 20 × 10^6^ cATMSCs if body weight was 10–20 kg and 30 × 10^6^ cATMSCs if body weight was 20–30 kg. Frozen vials of cATMSCs (10 × 10^6^ cell/mL) were hand-thawed and diluted in 50 mL of physiological saline serum and administered over 30 min through a peripheral intravenous (IV) cannula. The infusion was controlled by a veterinary. The dogs were monitored for 60 min following infusion and prior to being discharged. Any abnormal symptom, including laboratory parameters, were recorded by the investigators and considered as adverse events.

### 2.8. Statistical Methods

Each eye was considered as an independent sample and STT value was the primary endpoint. Variables were expressed as mean ± standard deviations (SD). Homogeneity between experimental (cATMSCs) and control (CsA) groups was tested by the independent samples Mann-Whitney U test for string variables and by the Chi-square test for categorical variables. Basal STT parameter was considered as the matched control. STT changes after cATMSCs infusion (for experimental group) or CsA+ artificial tears (for control group) treatment was analyzed using the nonparametric paired-group Wilcoxon signed-rank test for assessing treatment efficacy. Clinical evaluation data was analyzed employing a one-way ANOVA. Differences were considered significant when *p* < 0.05 (*). All analyses were carried out using SPSS 21.k.

## 3. Results

### 3.1. Characterization of Allogeneic cATMSCs by Flow Cytometry

**Figure 2:**
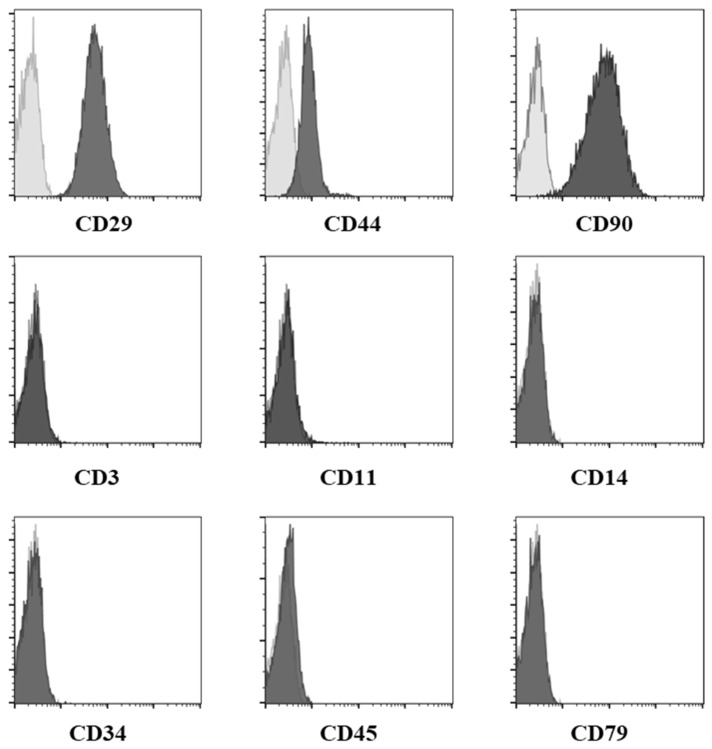
Expression of the different biomarkers on cATMSCs at passage 4 was assessed by flow cytometry. cATMSCs were positive for the identity panel of biomarkers (CD29, CD44, and CD90) and negative for the purity panel of biomarkers (CD3, CD11, CD14, CD34, CD45, and CD79). Legend: cAdMSC, canine adipose-tissue derived mesenchymal stem cell; Grey: negative controls. Black: stained cells.

The flow cytometry profiles of cATMSCs revealed a homogeneous cell population, positive to mesenchymal identity markers (CD29, CD44, and CD90), and negative for the expression of unrelated markers (MHC-II, CD3, CD11, CD14, CD34, CD45, and CD79 (Figure 2).

### 3.2. Systemic cATMSCs Administration

Fourteen dogs (twenty-six eyes) of different breeds, five males and nine females, with ages ranging from four to ten years old (7.00 ± 1.88 years) and weights from six to thirty-two kg (10.69 ± 7.31 kg) were enrolled in the study (mean ± SD for both variables, age and weight). All animals showed a history compatible with immune-mediated KCS for at least twelve months (2.36 ± 1.28 years) (Table 1). Twelve dogs (85.71%) presented bilateral KCS and two dogs (14.29%) unilateral KCS in the right eye. A control group of fourteen dogs was included. No statistically significant differences were observed between groups in any of the variables considered (Table 1).

Three dogs (five eyes) were withdrawn from the study due to different reasons: two animals needed immunosuppressive conventional treatments and one animal owner decided to withdraw from the study. Consequently, sample size was reduced to eleven dogs (twenty-one eyes).

Evaluation at day 180 was completed in nineteen out of twenty-one eyes that completed the study in day 45. There was no data from one dog (two eyes) as the owner did not visit the veterinary clinic again.

### 3.3. Clinical Evaluation and Efficacy Results

Basal clinical data were considered as normal (0–1) or mildly affected (2–4) at basal level (*n* = 21 eyes) (Table 2). Comparison between day 0, day 45, and day 180 was performed using one factor ANOVA. But no statistical significance was detected. Of note is the case of total ocular surface damage, that is the sum of values from: Nasal conjunctiva surface damage, temporal conjunctiva surface damage, and cornea surface damage. Data show a clear tendency to improvement, from 3.52 ± 2.79 mm/min at the beginning of treatment, to 2.29 ± 2.85 mm/min at day 45, and going down to 1.70 ± 1.83 mm/min at day 180. After one-factor ANOVA, no statistical significance was detected (*p* = 0.120).

Complete blood counts and serum chemistry profiles were within normal ranges. (Table S1). No side-effects were observed after systemic cATMSCs transplantation during the follow-up period.

**Figure 3:**
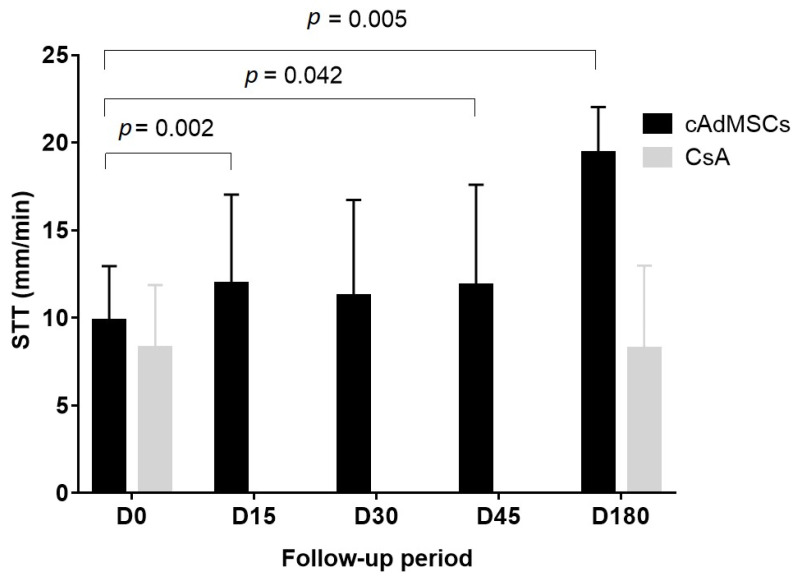
Graphical representation of the STT values in experimental (cATMSCs) and control (CsA) group during the study period. Bars represent the mean ± SD of STT measurements in each day of the study. Values were compared to basal status (D0). Legend: cAdMSC, canine adipose tissue-derived mesenchymal stem cell; CsA, cyclosporine A; D, day; SD, standard deviation; STT, Schirmer tear test; SD, standard deviation; STT, Schirmer tear test.

Basal STT values for the twenty-six eyes analyzed were lower than 15 mm/min (Table S2): twelve eyes showed STT values between 11–14 mm/min, ten eyes between 6–10 mm/min, and four eyes presented values lower or equal to 5 mm/min. Twenty-one eyes finished the follow-up on day 45 and STT values were significantly higher (*p* = 0.042, *n* = 21) compared to day 0. STT values increased significantly at days 15 (*p* = 0.002), 45 (*p* = 0.042) and 180 (*p* = 0.005) (Table 3 and Table S2, Figure 3). After cATMSCs treatment, dogs with basal STT between 11–14 mm/min maintained the significant increase in tear production up to day 180 (*p* < 0.05, *n* = 10). These animals needed only treatment with artificial tears. Nevertheless, dogs with basal STT < 11 mm/min needed CsA treatment at Day 45, so they were not followed up until day 180 (Table S2). Animals included in the control group and treated with CsA + artificial tears did not show a significant increase in tear production (*p* = 0.722, *n* = 28) from day 0 (STT, 8.32 ± 3.55 mm/min) up to day 180 (STT, 8.25 ± 4.74 mm/min) (Table S3).

Conjunctivitis and ocular surface evaluation were assessed 45 days after cATMSCs infusion and no statistically significant differences were observed (Table 2). Two dogs suffering from cutaneous atopy improved STT scores and cutaneous atopy.

## 4. Discussion

This study evaluates clinical and safety results after systemic administration of allogeneic cATMSCs in dogs with immune-mediated KCS. STT scores significantly increased at day 15, 45, and 180 of follow-up. Dogs treated with cATMSCs and with basal STT 11–14 mm/min needed only artificial tears at day 180, whilst dogs with basal STT <11 mm/min needed CsA treatment at day 45.

Systemic MSCs administration has been previously used for different pathological conditions in canine animal models showing not only safety but also efficacy [28,31,32]. According to our results, the systemic application of cATMSCs resulted in an increase in the STT score 15 days after the administration. It seems that the mechanisms of action of cATMSCs are more efficient when structural changes in the lacrimal gland are less profound. Consequently, cATMSCs should be applied in early stages of KCS, when the disease is not very advanced and still can be reverted.

Based on murine models of disease, it has been proposed that inflammation of the lacrimal gland triggers the recruitment of mesenchymal stem cells to the place of injury [33,34,35]. MSCs are stimulated by the proinflammatory cytokines and soluble factors released [26,36]. Exogenous MSCs might act by a paracrine effect, immunomodulating the inflammatory environment via secretion of soluble factors such as TGF-β, HGF, PGE2, and IDO [26,27]. By this paracrine effect, MSCs could restore a normal tears production and initiate the repair of the ocular surface. MSCs activity re-establishes the homeostasis of the affected lacrimal gland, restoring the normal tear production and growth factors secretion. This fact highlights their potential therapeutic use in the treatment of immune-mediated disease such as the KCS. Salivary gland has a limited ability to self-renew after injury by a process mediated by tissue stem cells [37]. Similarly, the lacrimal gland has significant regenerative potential, suggesting the presence of endogenous stem cells [38]. It is also possible that the exogenous MSCs stimulate the endogenous cells to provide an additional paracrine support or act by cell replacement as reported in other ocular diseases [39,40]. Nevertheless, tissue remodeling due to the deficient lacrimal gland activity (cornea damage, ulcers, scarring, neovascularization, etc.) develops with chronicity and is expected to be slower to revert, requiring longer periods to improve [41]. Anyhow, our results suggest that stem cell application can be helpful in the treatment of KCS symptoms.

We also described how two animals with concurrent cutaneous atopy improved their clinical signs, which could be related to the systemic application of cAdMSCs. Systemic implanted cATMSCs are able to exert their immunomodulatory role in the whole-body system (not just locally at the eye) by secreting paracrine factors [26], controlling several cell types related to the inflammatory response and also by recruiting other MSCs [35]. In consequence, the immunomodulatory response is not just boosted at the eye, but also extended to other inflammatory conditions present in the animal.

MSCs escape the normal process of alloantigen recognition due to their immune evasive characteristics [42]. Consequently, they are a promising new treatment for severe refractory autoimmune diseases [24,29,43,44,45]. Besides, treatment with allogeneic MSCs offers the advantage of being immediately available for therapy without the delay associated with the culture and expansion of autologous MSCs [42].

Common medication for immune-mediated KCS implies the use of corticosteroids or immunomodulatory drugs via topical eye application. After topical corticosteroids use, the drug acts locally, but it is also absorbed systemically through the conjunctiva and nasal or oral mucosa at a level of 1–35% of the dose [46]. This concentration can alter the hypothalamic-hypophyseal-adrenal axis. Treated dogs develop marked adrenal suppression followed by increased severity throughout the treatment period [47]. Consequently, dogs receiving chronic topical corticosteroid therapy should be checked for signs of iatrogenic hyperadrenocorticism and corticosteroid hepatopathy [48]. CsA treatment stops the aggression of the lacrimal gland and the inflammatory process, diminishes cornea and conjunctiva lesions, and restore eye lubrication. It must be administered twice a day, but if the treatment is interrupted, clinical signs will reappear [16]. Nevertheless, there are a number of canine KCS patients that do not respond to CsA treatment [14]. This fact is supported by our results in the control group where a high percentage of dogs did not improve their KCS measured as STT results. It is known that the stage of KCS at which CsA treatment is initiated is crucial in the response rate, given that the lacrimal glands in KCS become progressively deteriorated as the disease develops. Dogs with lower STT values were less responders to CsA treatment [13] and besides, CsA significantly increases tear production but in dogs with normal tear production [49]. We must highlight that half of the dogs included in the control group were diagnosed with severe or mild/moderate KCS, with low STT values and accordingly less responsive to CsA. Thus, new formulations proposed to suppress local immunity are pimecrolimus and tacrolimus, with similar effects to CsA. Likewise, they are daily topical drugs which effect disappears after medication withdrawn. The above-mentioned side-effects are in contrast with the lack of adverse events observed when treating KCS with cAdMSCs, thus highlighting the properties of MSCs as a new cell-based therapy, given their efficacy and safety.

Thus, in conclusion, systemic allogeneic cATMSCs application for canine early/subclinical KCS is an easily applied therapy with a significant improvement of tears production measured by STT and no associated adverse events. The STT increase lasted at least 180 days, without needing daily additional medication administration, thus suggesting it may be an alternative therapy to classical immunosuppressive treatments. We are aware that the design of this study has important limitations: fundamentally open label and limited number of patients (and eyes), which could affect the evaluation of the results. Longer follow-up periods and measurements of tears composition to monitor growth factors restoring secretion might be also needed.

## Figures and Tables

**Table 1:** Baseline demographic data.

Dog Characteristics	cAdMSCs (*n* = 14)	Control (*n* = 14)	Mann-Whitney *U* Test *p* Value	Chi-Square *p* Value
Age (Years; mean ± SD)	7.00 ± 1.88	6.71 ± 1.90	0.701	
Weight (Kg; mean ± SD)	10.69 ± 7.31	10.32 ± 6.18	0.635	
KCS history (Years; mean ± SD)	2.36 ± 1.28	2.23 ± 1.17	0.734	
Sex (*n* (%))				1.000
Male	5 (35.70)	9 (64.30)		
Female	9 (64.30)	5 (35.70)		
Breed (*n* (%))				0.766
British bulldog	1 (7.10)	-		
Cocker spaniel	-	1 (7.14)		
Crossbreed	3 (21.50)	2 (14.29)		
French bulldog	1 (7.10)	3 (21.43)		
Golden	-	1 (7.14)		
Ponter	-	1 (7.14)		
Pug	1 (7.10)	1 (7.14)		
Schnauzer	1 (7.10)	-		
Shih-Tzu	3 (21.50)	2 (14.29)		
Weim	1 (7.10)	-		
West Highland White Terrier	1 (7.10)	1 (7.14)		
Yorkshire Terrier	2 (14.40)	2 (14.29)		

cAdMSC, canine adipose tissue-derived mesenchymal stem cell; KCS, keratoconjunctivitis sicca; SD, standard deviation.

**Table 2:** Clinical data at baseline and follow-up in the cATMSCs group. Eye characteristics were classified as normal (0–1), mildly affected (2–4), moderately affected (5–7), and severely affected (8–10). Ocular surface damage in the nasal conjunctiva, the temporal conjunctiva, and the cornea was graded as absent (0), mild (1), moderate (2), or severe (3) based on the results obtained after Rose Bengal staining. Total ocular surface damage result was the addition of the three previous values (0–9).

	Day 0 (Visit 1) *n* = 21	Day 45 (Visit 4) *n* = 21	Day 180 (Visit 5) *n* = 10
**Eye characteristics (mean ± SD)** (range 0–10)			
Corneal vascularization	2.05 ± 2.82	2.14 ± 2.97	2.10 ± 2.56
Overlying epithelium	1.48 ± 2.87	1.48 ± 2.87	1.30 ± 2.21
Palpebral closing limitation	1.00 ± 2.43	1.00 ± 2.43	0.50 ± 0.97
Subepithelial oedema	2.52 ± 3.67	2.67 ± 3.86	2.70 ± 2.91
Abrasion/Ulcers	0.00 ± 0.00	0.00 ± 0.00	0.00 ± 0.00
Blepharospasm	1.24 ± 1.95	1.00 ± 2.39	0.00 ± 0.00
Pigmentary keratitis	2.38 ± 3.53	2.24 ± 3.48	2.40 ± 2.80
Keratohelcosis	0.00 ± 0.00	0.00 ± 0.00	0.00 ± 0.00
**Ocular surface damage (mean ± SD)** (range 0–3)			
Nasal conjunctiva surface damage	1.14 ± 1.11	0.67 ± 1.02	0.30 ± 0.48
Temporal conjunctiva surface damage	1.00 ± 1.14	0.48 ± 0.93	0.30 ± 0.48
Cornea surface damage	1.38 ± 1.40	1.14 ± 1.31	1.10 ± 1.29
Total ocular surface damage	3.52 ± 2.79	2.29 ± 2.85	1.70 ± 1.83
Conjunctivitis type (*n* (%))	*n* = 20	*n* = 17	*n* = 10
Mucoid	13 (65.00)	7 (41.20)	2 (20.00)
Muco-purulent	4 (20.00)	2 (11.80)	0 (0.00)
Transparent serous	1 (5.00)	8 (47.00)	6 (60.00)
Serous	0 (0.00)	0 (0.00)	2 (20.00)
Purulent	2 (10.00)	0 (0.00)	0 (0.00)

**Table 3:** Statistic data of STT values (expressed in mm/min) in experimental (cATMSCs) and control (CsA) group during the study period.

	Day 0 *n* = 21	Day 15 *n* = 21	Day 30 *n* = 21	Day 45 *n* = 21	Day 180 *n* = 10
STT (Mean ±SD)	9.71 ± 3.34	11.95 ± 5.08	11.24 ± 5.50	11.86 ± 5.74	19.40 ± 2.63
Wilcoxon signed-rank test (vs. Day 0)		*p =* 0.002	*p =* 0.294	*p =* 0.042	*p =* 0.005

## Supplementary Materials

The following are available online at www.mdpi.com/article/10.3390/jcm10245981/s1, Table S1: Basal biochemistry and haematological data in the cATMSCs group, Table S2: STT values at Day 0, 15, 45 and 180 in the cATMSCs group, Table S3: STT values at Day 0 and 180 in the control group.
