# Supplementary material for: Systemic Treatment of Immune-Mediated Keratoconjunctivitis Sicca with Allogeneic Stem Cells Improves the Schirmer Tear Test Score in a Canine Spontaneous Model of Disease
Source: J Clin Med. 2021 Dec 20;10(24):5981. doi: 10.3390/jcm10245981 (PMC8709250; doi:10.3390/jcm10245981)
Supplement: Supplementary file 1 [file jcm-10-05981-s001.zip › jcm-1475829-supplementary.pdf]

Journal of
Clinical Medicine

Table S1: Basal biochemistry and haematological data in the cATMSCs group.

**Animals (n = 14)**	**Reference values**	**Mean ± SD**
Haematocrit (%)	37.00 - 55.00	49.85 ± 8.12
Haemoglobin (g/dl)	12.00 - 18.00	16.42 ± 2.84
Erythrocytes (units/µl)	5,500,000.00 - 8,500,000.00	6,674,615.38 ± 1,045,543.04
MCV (fl)	60.00 - 77.00	74.70 ± 3.52
MCH (pg)	19.00 - 25.00	24.56 ± 1.01
MCHC (g/dl)	32.00 - 36.00	32.89 ± 1.22
Platelets (units/µl)	200,000.00 - 500,000.00	267,083.33 ± 75,883.23
Leucocytes (units/µl)	6,000 - 17,000	8,580.77 ± 3,009.62
Eosinophils (%)	0.00 - 10.00	4.15 ± 2.34
Basophils (%)	0.00 - 1.00	0.15 ± 0.38
Lymphocytes (%)	12.00 - 30.00	18.92 ± 5.33
Monocytes (%)	3.00 - 10.00	5.31 ± 2.84
Bands (%)	0.00 - 3.00	0.00 ± 0.00
Segmented (%)	60.00 - 77.00	71.46 ± 6.63
Eosinophils (units/µl)	100.00 - 1,250.00	353.57 ± 205.55
Basophils (units/µl)	0.00 - 200.00	9.7 ± 23.71
Lymphocytes (units/µl)	1,000.00 - 4,800.00	1,598.96 ± 662.75
Monocytes (units/µl)	150.00 - 1,350.00	438.45 ± 238.32
Bands (units/µl)	0.00 - 300.00	0.00 ± 0.00
Segmented (units/µl)	3,000.00 - 11,500.00	6,180.08 ± 2,344.80
Reticulocytes (%)	0.50 - 2.00	0.72 ± 0.33
Reticulocytes ($10^3/mm^3$)	(0.00 - 70.00)	53.95 ± 24.07
Ultra-sensitive C-reactive protein (mg/l)	< 0.15	0.05 ± 0.03
Creatinine/Serum (mg/dl)	< 1.80	0.92 ± 0.15
Total protein/Serum (g/l)	54.00 - 71.00	69.36 ± 6.10
Total globulin/Serum (g/l)	15.00 - 35.00	37.57 ± 7.65
Albumin/Serum (g/l)	25.00 - 40.00	31.79 ± 4.28
Albumin/Globulin ratio		0.89 ± 0.22
Gamma-GT (U/l)	< 15.00	9.17 ± 9.77
Alkaline phosphatase/Serum (U/l)	< 200.00	84.64 ± 106.63

cAdMSC, canine adipose-tissue derived mesenchymal stem cell; MCH, mean corpuscular haemoglobin; MCHC, mean cell haemoglobin concentration; MCV, mean corpuscular volume; SD, standard deviation.

Table S2: STT values at Day 0, 15, 45 and 180 in the cATMSCs group.

			STT				**Treatment**
Eye	D -15	D 0	D 15	D 30	D 45	D 180	After D180
A001R	10	14	17	12	14	20	Hylo Gel®
A001L	15	13	15	11	16	18	Hylo Gel®
A002R	13	13	19	9	16	21	Hylo Gel®
A002L	13	14	19	12	14	21	Hylo Gel®
A003R	20	13	13	23	21	21	Hylo Gel®
A003L	13	12	20	23	23	23	Hylo Gel®
A004R	8	12	16	15	5	14	Hylo Gel®
A004L	11	14	17	13	16	21	Hylo Gel®
A005R	0	10	8	8	8	No data	No data
A005L	0	6	4	5	5	No data	No data
A007R	11	11	10	12	16	18	Hylo Gel®
A007L	9	11	13	9	10	17	Hylo Gel®
B002R	11	8	10	4	6	No data	CsA
B002L	10	7	10	4	4	No data	CsA
B003R	16	9	16	16	18	No data	CsA
B003L	8	5	8	8	7	No data	CsA
B004R	9	7	8	13	12	No data	CsA
B004L	8	7	7	13	13	No data	CsA
B005L	17	7	10	15	13	No data	CsA
B006R	5	5	5	5	5	No data	CsA
B006L	10	9	9	9	10	No data	CsA

cAdMSC, canine adipose tissue-derived mesenchymal stem cell; CsA, cyclosporine A; D, day; STT, Schirmer Tear Test.

Table S3: STT values at Day 0 and 180 in the control group.

	STT		Treatment
Eye	D 0	D 180	After D180
CTR01L	12	14	CsA + Hylo Gel®
CTR01R	13	14	CsA + Hylo Gel®
CTR02L	14	15	CsA + Hylo Gel®
CTR02R	12	13	CsA + Hylo Gel®
CTR03L	13	15	CsA + Hylo Gel®
CTR03R	11	16	CsA + Hylo Gel®
CTR04L	14	16	CsA + Hylo Gel®
CTR04R	12	11	CsA + Hylo Gel®
CTR05L	9	6	CsA + Hylo Gel®
CTR05R	7	6	CsA + Hylo Gel®
CTR06L	5	6	CsA + Hylo Gel®
CTR06R	7	8	CsA + Hylo Gel®
CTR07L	10	8	CsA + Hylo Gel®
CTR07R	9	7	CsA + Hylo Gel®
CTR08L	10	9	CsA + Hylo Gel®
CTR08R	10	12	CsA + Hylo Gel®
CTR09L	1	0	CsA + Hylo Gel®
CTR09R	3	2	CsA + Hylo Gel®
CTR10L	8	9	CsA + Hylo Gel®
CTR10R	5	4	CsA + Hylo Gel®
CTR11L	5	4	CsA + Hylo Gel®
CTR11R	7	8	CsA + Hylo Gel®
CTR12L	6	8	CsA + Hylo Gel®
CTR12R	6	3	CsA + Hylo Gel®
CTR13L	7	4	CsA + Hylo Gel®
CTR13R	8	8	CsA + Hylo Gel®
CTR14L	2	0	CsA + Hylo Gel®
CTR14R	7	5	CsA + Hylo Gel®

CsA, cyclosporine A; D, day; STT, Schirmer Tear Test
